# Evaluation of the quality of informed consent in a vaccine field trial in a developing country setting

**DOI:** 10.1186/1472-6939-9-15

**Published:** 2008-09-30

**Authors:** Deon Minnies, Tony Hawkridge, Willem Hanekom, Rodney Ehrlich, Leslie London, Greg Hussey

**Affiliations:** 1 South African Tuberculosis Vaccine Initiative, Institute of Infectious Diseases and Molecular Medicine & School of Child and Adolescent Health, University of Cape Town, Cape Town, South Africa; 2 School of Public Health and Family Medicine, Faculty of Health Sciences, University of Cape Town, Cape Town, South Africa

## Abstract

**Background:**

Informed consent is an ethical and legal requirement for research involving human participants. However, few studies have evaluated the process, particularly in Africa.

Participants in a case control study designed to identify correlates of immune protection against tuberculosis (TB) in South Africa. This study was in turn nested in a large TB vaccine efficacy trial.

The aim of the study was to evaluate the quality of consent in the case control study, and to identify factors that may influence the quality of consent.

Cross-sectional study conducted over a 4 month period.

**Methods:**

Consent was obtained from parents of trial participants. These parents were asked to complete a questionnaire that contained questions about the key elements of informed consent (voluntary participation, confidentiality, the main risks and benefits, etc.). The recall (success in selecting the correct answers) and understanding (correctness of interpretation of statements presented) were measured.

**Results:**

The majority of the 192 subjects interviewed obtained scores greater than 75% for both the recall and understanding sections. The median score for recall was 66%; interquartile range (IQR) = 55%–77% and for understanding 75% (IQR = 50%–87%). Most (79%) were aware of the risks and 64% knew that they participated voluntarily. Participants who had completed Grade 7 at school and higher were more likely (OR = 4.94; 95% CI = 1.57 – 15.55) to obtain scores greater than 75% for recall than those who did not. Participants who were consented by professional nurses who had worked for more than two years in research were also more likely (OR = 2.62; 95% CI = 1.35–5.07) to obtain such scores for recall than those who were not.

**Conclusion:**

Notwithstanding the constraints in a developing country, in a population with low levels of literacy and education, the quality of informed consent found in this study could be considered as building blocks for establishing acceptable standards for public health research. Education level of respondents and experience of research staff taking the consent were associated with good quality informed consent.

## Background

Informed consent is an ethical and legal requirement for research involving human participants [1]. Guidelines as contained in the Declaration of Helsinki, the Nuremburg Code and the Belmont Report [2], and the Council for the International Organizations of Medical Research [3] have become accepted tools used by Institutional Review Boards (IRBs), the bodies responsible for ethical review of research proposals, in approving such research. The International Convention for Harmonization's initiative on Good Clinical Practice (GCP) [4] is considered to be the quality standard for the conduct of clinical trials in humans. Accredited courses for investigators of clinical trials are based on these GCP guidelines. Medical doctors, professional nurses, and other health professionals who conduct research on behalf of principal investigators frequently do these courses as part of their training.

In most countries research ethics are regulated by statutory bodies [5,6]. IRBs of sponsor organizations and Research Ethics Committees (RECs) of academic institutions are mandated by their stakeholders to protect participants.

However, unethical research practices occur not uncommonly and appear to have been accepted and even perpetuated by IRBs [7-9]. Some authors have reservations about the capabilities of IRBs in developing countries, suggesting that they may be made up by inadequately skilled members [10]. London [11] proposed ways to improve the regulation of ethics practices in developing countries, which included proper research ethics training of IRB members and independent monitoring of research activities.

Studies of the quality of informed consent in medical practice have found this to be poor [12-14]. Similar shortcomings in consent quality in research have been found [15-17], particularly in developing countries, where concepts such as "voluntary participation [18,20]", "randomization [19]" and "benefits and risks [20]" may have variable interpretations. Large scale research projects are increasingly being conducted in these settings. The informed consent process poses several challenges, logistic as well as ethical, since failures of informed consent may result in the violations of participants' human rights. In South Africa, where the rights of the research participant are explicitly protected in the Constitution [6], it is essential that this understanding of participants' rights be tested in the appropriate research environment. Poverty, disease, lack of education, hardship, submissiveness, the effects of war, famine, pandemics, and social insecurity prevalent in developing countries all make participants more vulnerable to research exploitation.

### Purpose & objectives

This secondary study, the "consent study", aimed to evaluate the quality of informed consent in a primary case control study (the Immunology Study) of immune correlates of protection against severe childhood TB nested inside a randomized controlled trial vaccine trial [Bacille Calmette-Guerrin (BCG) Study]. A total of 5467 children were enrolled into the Immunology Study from 2001 to 2004. These studies were implemented in Worcester, a rural setting in the Western Cape Province of South Africa. Participants in the primary immunology case control study underwent collection of blood between 8 and 14 weeks of age, after written consent had been obtained from their parents. The consent procedure was concluded by means of signing a consent form.

The objectives of this consent study were:

a. To determine study participants' recall and understanding of items discussed during the consent procedure for the immunology study;

b. To establish whether certain participant and study-related factors were associated with the quality of informed consent; and

c. To describe the association between the quality of informed consent and participants' knowledge of their health rights.

## Methods

The study was a cross-sectional study conducted over four months

### Population and sampling

The population for the consent study was drawn from mothers who gave informed consent for the participation of their children in the Immunology Study. Recruitment for the Immunology Study took place from March to June 2004 in the form of a team visiting district health facilities according to a fixed schedule. Enrolment and phlebotomy were preceded by a booking session at which an information pamphlet about the Immunology study was handed out. Informed consent was conducted in the first language of the mother (either Afrikaans or Xhosa) by trained interviewers.

For the consent study, all mothers attending clinics where the language of communication was predominantly Xhosa, and every fourth mother attending Afrikaans-language clinics were approached to participate in the consent study.

Dedicated nursing staff conducted the interview within one hour of mothers' consent to their infant's participation in the Immunology Study.

### Measurement and data collection

The quality of informed consent was determined by measuring the recall and understanding of informed consent using a questionnaire specifically designed for this study (see additional file 1). The questionnaire contained nine questions dealing with the basic facts of the Immunology Study. Participants were expected to select the correct answer from a choice of three possible answers for each of the questions. One of the answers was an exact reflection of the information in the consent document, which, if selected, was taken as an indicator of correct recall. For the understanding assessment, participants were expected to select the appropriate interpretations for a total of eight statements offered. This was taken as an indication of the extent to which participants' decisions were based on understanding. Although there is some inevitable overlap between recall and understanding, the type of questions allowed broad categorization into two separate scales for recall and understanding. Participants were requested to complete the questionnaire in writing while consent study staff provided assistance with the interpretation of the questions.

### Statistical methods

The results on the data form were captured, processed and analysed using Stata version 6 [21]. Correct scores for recall and understanding were totaled for individual participants and summary statistics were calculated. Total scores for individual recall and health rights questions were also calculated. Logistic regression analyses were performed to model the effect of maternal age, education, access to telephones, language preference and research experience of the professional nurse on the recall and understanding scores.

### Ethical considerations

The Research Ethics Committee of the University of Cape Town Health Sciences faculty approved both the Immunology and Consent Studies. Mothers who had consented to participation into the Immunology Study were referred to the nurse for the Consent Study in a separate room. As part of the consent procedure, the nurse handed the mother an information sheet written in simple language explaining the Consent Study, and asked the mother to read it. After verifying that the mother understood the contents of the consent letter, she was then asked to participate. If she accepted, the nurse asked the mother to acknowledge by signing and dating a copy of the letter. This copy was kept for record purposes.

## Results

**Figure 1:**
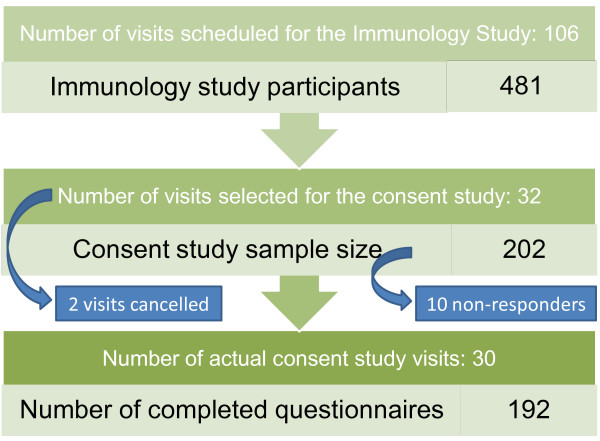
**Schema to show how participants were selected for the consent study**. Of the 106 Immunology Study visits ("phlebotomy clinics") scheduled for the study period, 32 were selected through systematic sampling. A total of 481 participants enrolled for the Immunology Study during the corresponding period. Of these, 202 were eligible to participate in the consent study. Eventually, 192 participants completed the consent study questionnaire.

Four hundred and eighty-one participants from 106 clinic visits to 22 primary healthcare facilities (clinics) in the study area were enrolled into the Immunology Study. Of the 32 visits which were selected for the Consent Study, two visits were cancelled. A total of 202 Immunology Study participants were referred to the Consent Study staff. One-hundred-and-ninety-two (192) mothers completed the questionnaires for the Consent Study, resulting in a response rate of 95.0% (Figure 1). The non-responders were made up as follows: three left the clinic before the Consent Study team had started enrollments, four could not be enrolled because of an accidental language mismatch and a further three used spoilt versions of the questionnaire. The demographic characteristics of the sample are listed in Table 1.

**Table 1:** Demographic characteristics (n = 192)

**Participant aspect**		**Consent Study**
Age (years):	median (range)	26 (16–44)
Language:	% Afrikaans	56.8
	% Xhosa	43.2
Level of education:	(% Grade 6 or less)	23.4
	(% Grade 7 to 11)	42.8
	(% Grade 12 or higher)	33.9
Access to a telephone:	(%)	57.9
Attendance per clinic:	median (range)	8 (1–15)

**Figure 2:**
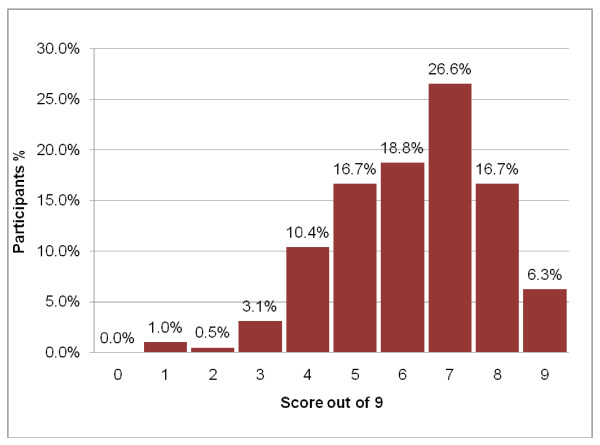
Distribution of the results (scores out of 9) of the recall test.

**Figure 3:**
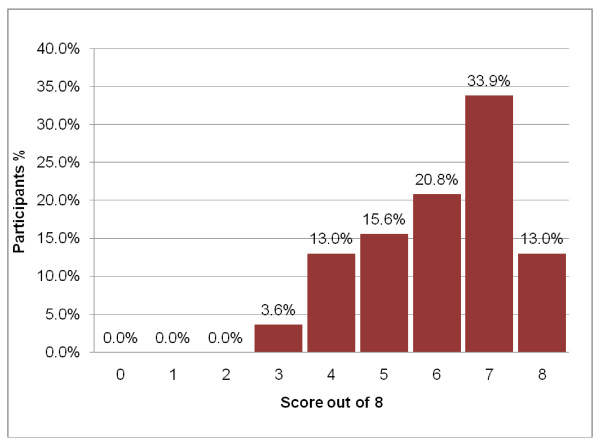
Distribution of the results (scores out of 8) of the understanding test.

Participants obtained a median score of 66.7% (range 11.1% – 100.0%) in the recall and 75.0% (range 37.5% – 100%) in the understanding sections. Both scores were skewed towards the left (Figures 2, 3). Although only 12 (6%) and 25 (13%) of participants respectively had all the answers to the recall and understanding questions correct, the majority of participants obtained scores in the "75% or greater" category. Only 3 (1.6%) obtained a score of less than 25% for the recall test and none had two or fewer correct out of eight understanding questions. As can be seen from Table 2, higher levels of recall scores were positively associated with higher levels of understanding levels scores (Spearman correlation coefficient = 0.37, p = 0000).

**Table 2:** Relationship between recall and understanding score (n = 192)

	**Understanding n (%)**			
	High*	High medium*	Low medium*	Low*

Recall n (%)				
High*	76 (80.0%)	17 (17.9%)	2 (2.1%)	0 (0.0%)
High medium*	39 (57.3%)	25 (36.8%)	4 (5.9%)	0 (0.0%)
Low medium*	14 (53.9%)	11 (42.3%)	1 (3.9%)	0 (0.0%)
Low*	1 (33.3%)	2 (66.7%)	0 (0.0%)	0 (0.0%)

* High: [≥ 75%]; High medium: [≥ 50% & < 75%]; Low medium: [≥ 25% & < 50%]; Low: [< 25%]

**Figure 4:**
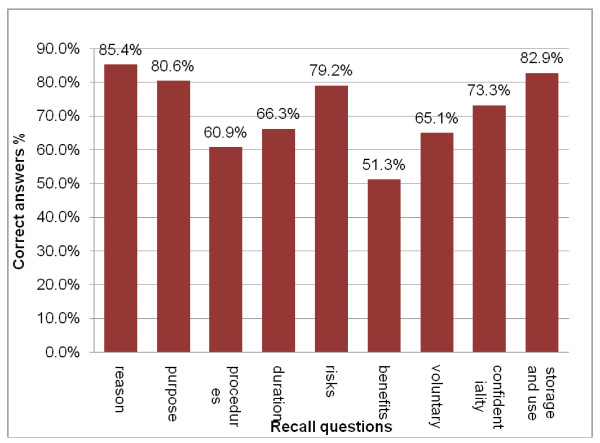
**Overall results of the recall test**. The percentage of participants who provided the correct answer to each of the recall questions.

Recall of questions: Four out of the nine recall questions elicited more than 75% correct answers (Table 3), and another four elicited between 50% and 75% correct answers. The question used to test whether participants knew the reason for the clinic visit received the highest proportion (85.4%) of correct answers (Figure 4). Notably, only 36.5% of participants knew that there were no immediate benefits. Otherwise, correct responses exceeded 60% for other questions.

**Table 3:** Results of quality of informed consent assessment: Recall section

**1. I have been asked to attend the clinic today so that (n = 192):**	
*My baby can participate in a research study*	164 (85.4%)
My baby can receive expert treatment.	14 (7.3%)
My baby can receive routine health care	14 (7.3%)

**2. The purpose of the research study is to (n = 191):**	

Test for protection against tuberculosis in my baby's blood.	154 (80.6%)
Test for tuberculosis in my baby's blood	35 (18.3%)
Test for HIV in my baby's blood	2 (1.1%)

**3. Research staff wants to enroll my baby into the research study so that (n = 189):**	

*They can collect blood from my baby*	115 (60.9%)
They can inject my baby with BCG	40 (21.1%)
They can test my baby for TB or HIV	34 (18.0%)

**4. The total amount of time my baby will be expected to participate in the study is (n = 190):**	

*1 day*	126 (66.3%)
2 to 3 years	45 (23.7%)
8 to 14 weeks	19 (10.0%)

**5. The most common risk involved when blood had been collected from my baby is (n = 192):**	

*My baby may suffer very slight scarring and some oozing*	152 (79.2%)
My baby can become infected with TB or HIV	35 (18.2%)
My baby can loose too much blood	5 (2.6%)

**6. The benefits available to me and my baby for participating in the study are (n = 191):**	

My baby will be protected against TB	98 (51.3%)
*There are no immediate benefits*	70 (36.7%)
My baby and I will get better treatment at clinics	23 (12.0%)

**7. If I didn't want to participate in this study, I could withdraw and (n = 189)**	

*My baby and I would suffer no loss at all*	123 (65.1%)
My baby and I will be treated differently by research and clinic staff	47 (24.9%)
My baby and I would be denied access to health services at this clinic	19 (10.0%)

**8. My baby's personal details will never be linked with her blood because (n = 191)**	

*Numbers with barcodes will be used to keep bloods anonymous*	140 (73.3%)
Highly trained research staff will keep information secret	40 (20.9%)
Clinic staff will be sure not to give information to the research staff	11 (11.8%)

**9. The blood of my baby that will be frozen and stored will be used (n = 187)**	

*For other tests concerning protection against TB*	155 (82.9%)
For all kinds of research in other countries	29 (15.5%)
For HIV testing	3 (1.6%)

Correct answers based on information provided as part of the consent process in italics and listed first here, but in normal print and distributed randomly in the field questionnaire

The final logistic regression model obtained for recall scores of 75% or greater included experience of research nurse and education level of the participant. Participants who had completed Grade 7 and higher at school were more likely (OR = 4.94, 95% CI = 1.57–15.55) to obtain a minimum of 75% in the recall test compared to participants who did not progress that far in education. Also, consent obtained by nurses with more than two years research experience resulted in an almost three times (OR = 2.62, 95% CI = 1.35 – 5.07) the odds of scoring at least 75% compared to nurses with less than two years of experience.

Understanding of questions: Most participants gave the expected answers (Table 4) for all understanding questions. Although 87.5% of participants understood that the development of a bruise did not warrant a call to the police, a relatively low percentage (66.2%) knew that they could discuss the bruise with the nurse at the clinic. The logistic regression model obtained for understanding scores of 75% and greater indicated that participants who were older than the median ages of 26 years were more likely (95% CI = 1.15 – 4.07) to obtain high scores than those younger than 26 years. Level of education was not associated with understanding in this model.

**Table 4:** Results of quality of informed consent assessment: Understanding section

**I agreed to enrol my child in this study because**			
	**True**	**False**	**Not sure**

My child might get better treatment	44.8	*52.1*	3.1
I want doctors to help learn more about TB	*88.5*	4.7	6.8

**I've decided to enrol my baby in the study**			

Even though my baby will receive no extra treatment	*73.4*	19.3	7.3
Because I knew I would receive a toiletries hamper	13.0	*80.7*	6.3

**If my baby gets a bruise from the blood test, I should**			

Contact the police	5.7	*87.5*	6.8
Speak to the nurse at the clinic	*68.2*	30.7	1.1
Go to the doctor at his private surgery	22.4	*66.2*	11.4

**If I was given the choice to participate again, I would**	*90.6*	3.1	6.3

Correct interpretation, according to the information provided during the consent process, in bold type here, but in normal print in the field questionnaire.

**Figure 5:**
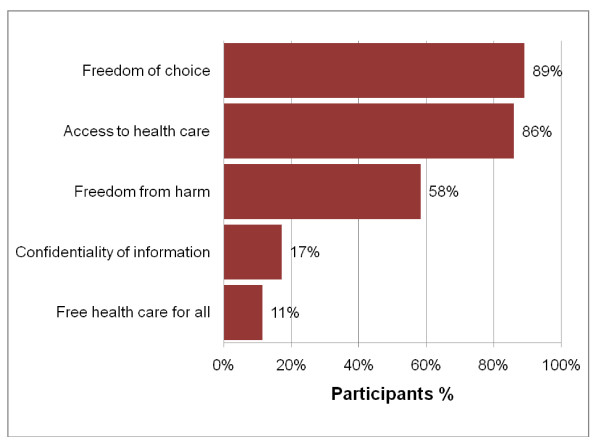
**Knowledge of health rights**. The percentage of participants who provided the correct answer to each of the health rights questions.

Knowledge of health rights as contained in the South African Constitution (see figure 5): The majority of participants were aware of their rights regarding access to health care, freedom of choice and freedom from harm. However, confidentiality of information and free health care for all were mistaken as rights by most participants. The overall score for health rights was not linearly correlated with either the recall or the understanding assessment (Spearman's correlation coefficient not greater than |0.10| and p = 0.96 and 0.41, respectively). Individual scores for each of the health rights also had no association with the recall scores, and all but one of the understanding scores. The knowledge of right to freedom of choice was associated with the understanding score (OR = 2.59, 95% CI = 1.06 – 6.34).

## Discussion

The finding that most participants obtained scores in excess of 75% for understanding and 66% for recall indicates that the general quality of informed consent was encouraging. Almost all participants felt that they would participate again in a similar study if they were given the choice. These results are similar to both qualitative and quantitative findings in previous studies conducted in developing countries [18,20,22].

The proportion of participants with low scores for quality of informed consent was small and lower even than that found in some studies in industrialized countries [16,23]. However, due to differences in methodologies, no specific comparison is attempted. The understanding assessment result supports earlier suggestions [24] that low levels of education need not hinder participants' ability to understand consent concepts.

It is reassuring that the two language groups had similar results, as there had been concerns in the trial that consent quality would be different between the language groups. The two languages broadly represent two different race groups in South Africa, which could be associated with differences in perception of social services such as health care. Barsdorf & Wassenaar [25] have shown that Black participants, known to have suffered most from the injustices of the apartheid regime, had poorer perception of voluntariness than their White and Indian counterparts. These differences in perceptions of research between race groups were not demonstrated in this study.

Some limitations in the measurement method are evident. The study did not assess the ability of participants to retain information for longer periods of time (i.e. days, weeks or months) after their enrolment into the Immunology Study. Moreover, the questions posed for understanding could not cover all the emotional and intellectual aspects involved when giving consent. Access to a telephone or cell-phone may not be the best proxy for socioeconomic status as it may be too indirect and focuses only on the "economic" part of status. There is also an unquantifiable overlap between recall and understanding. Nonetheless, despite the recognized difficulties of measuring understanding with informed consent procedures, the findings of this study pointed to the need to employ methods to enhance informed consent quality in less educated participants, such as the use of audiovisual aids.

The following are required for a good consent quality: a much greater focus on the consent process, the assurance that participants possess the required level of education (in South Africa Grade 7 is considered minimum), that voluntary participation is encouraged and that confidentiality is secured. In addition, an abridged form of self-assessment by participants, such as the type described in this study, could improve the general understanding of research concepts, such as voluntary participation and confidentiality by prospective research participants. A second and third attempt at explaining some of the more difficult concepts might help to ensure better recall and understanding of information.

We suggest that research ethics committees should insist on periodic reports on consent quality and efforts to improve quality where appropriate as part of their responsibility to protect the public against unethical research practices. The Consent Study focused on the evaluation of the quality of informed consent by using a self-administered questionnaire. Although level of education and experience of the research staff were predictors of good quality of consent in this study, researchers would need to identify the predictors of good quality consent in their studies. Although this study chose to weight recall and understanding equally, consideration could be given to whether specific questions, such as those pertaining to perceived benefits or ability to withdraw, should have higher weight in deciding whether participant consent is adequate.

The Immunology Study was conducted in a rural district known for its low socio-economic status, high unemployment and high prevalence of diseases of poverty. Our experience is that participants are generally research naïve, and because of problems of staff shortage and scarce resources in the health services, frequently confuse research activities with health care delivery, and therefore welcome the attention of better resourced research initiatives. This renders them a particularly vulnerable research population. Given this context, the findings of the Consent Study can be considered encouraging, in particular because it appears as if most participants had made informed choices about their participation in the Immunology Study. Most participants had medium to high understanding of the study. Misunderstanding about perceived benefits is understandable, but it could also mean that participants made the decision to participate in the Immunology Study because of a belief that the benefits were greater than they actually were.

## Conclusion

This study should add to the sparse literature dealing with the quantitative evaluation of informed consent in a developing country situation, and encourage commentary and further research in this important area of research ethics, including further exploration into the question of acceptable standards for quality of informed consent.

While developed country-based sponsors are likely to be centrally concerned with the scientific question, and research ethics committees about the general ethical conduct of proposed research, principal investigators in developing countries should consciously consider attaining good quality informed consent as a key component of their research proposals.

In this study, good quality informed consent was associated with higher levels education of respondents and experience of staff obtaining the consent. Many research studies are conducted in developing countries in similar settings, i.e. where there are language and cultural differences between researchers and participants, low education and socio-economic status and limited resources. There is therefore no reason that the same quality of informed consent should not be achieved in other parts of Southern Africa and abroad.

## Competing interests

The authors declare that they have no competing interests.

## Authors' contributions

DM conceived the study in conjunction with TH, GH and WH.  DM designed the study and its instruments with assistance from TH, RE and LL.  DM implemented the study, assisted by TH, GH and WH.  DM performed the analysis with the assistance of TH and RE.  DM wrote the manuscript with the assistance of TH, RE and GH.  All authors read and approved the final manuscript

## Pre-publication history

The pre-publication history for this paper can be accessed here:



## Supplementary Material

Additional file 1 **Questionnaire used as data collection tool in the consent study.** Click here for file
