# Supplementary material for: Evaluation of the quality of informed consent in a vaccine field trial in a developing country setting
Source: BMC Med Ethics. 2008 Sep 30;9:15. doi: 10.1186/1472-6939-9-15 (PMC2575196; doi:10.1186/1472-6939-9-15)
Supplement: Additional file 1 — Questionnaire used as data collection tool in the consent study. [file 1472-6939-9-15-S1.doc]

Serial number

**QUALITY OF CONSENT EVALUATION QUESTIONNAIRE**

| Study baby T-number |  | Participant name |  | | | | |
| --- | --- | --- | --- | --- | --- | --- | --- |
| Participant date of birth |  | Participant address |  | | | | |
| Date of interview |  | Language spoken at home eg A for Afrikaans |  | Education:  Eg. G 8 for Grade 8 or St 8 for Standard 8 |  | Tel |  |
|  |  |  |  |  |  | Cell |  |

- 1. Select the ONE option that best completes the statement regarding the study you were asked to consent to earlier today (Correct answer in red – for illustration only. For survey all options will appear identical in print.)

1. I have been asked to attend the clinic today so that:
   1. *My baby can receive expert treatment.*
   2. *My baby can participate in a research study*
   3. *My baby can receive routine health care*
2. The purpose of the research study is to:
   1. *Test for protection against tuberculosis in my baby’s blood.*
   2. *Test for tuberculosis in my baby’s blood*
   3. *Test for HIV in my baby’s blood*
3. Research staff wants to enroll my baby into the research study so that:
   1. *They can test my baby for TB or HIV*
   2. *They can collect blood from my baby*
   3. *They can inject my baby with BCG*
4. The total amount of time my baby will be expected to participate in the study is:
   1. *2 to 3 years*
   2. *8 to 14 weeks*
   3. *1 day*
5. The most common risk involved when blood had been collected from my baby is:
   1. *My baby can become infected with TB or HIV*
   2. *My baby may suffer very slight scarring and some oozing*
   3. *My baby can loose too much blood*
6. The benefits available to me and my baby for participating in the study are:
   1. *There are no immediate benefits*
   2. *My baby will be protected against TB*
   3. *My baby and I will get better treatment at clinics*
7. If I didn’t want to participate in this study, I could withdraw and
   1. *My baby and I would be denied access to health services at this clinic*
   2. *My baby and I will be treated differently by research and clinic staff*
   3. *My baby and I would suffer no loss at all*
8. My baby’s personal details will never be linked with his / her blood because
   1. *Numbers with barcodes will be used to keep bloods anonymous*
   2. *Highly trained research staff will keep information secret*
   3. *Clinic staff will be sure not to give information to the research staff*
9. The blood of my baby that will be frozen and stored will be used
   1. *For all kinds of research in other countries*
   2. *For HIV testing*
   3. *For other tests concerning protection against TB*

# B. State whether you agree with the statements below by ticking off the appropriate box next to each statement.

Yes No Don’t

know

1. The procedures done on my baby in this study are:
   1. Safe and practically harmless………………………….   
   2. Dangerous and harmful……………….……………..…   
2. My baby’s blood is going to be used to
   1. help develop a blood test for TB……………………...   
   2. determine whether my baby has TB…………………..   
3. My baby’s name is written on all the blood tubes…………..   
4. I agreed to enroll my child in this study because:
   1. My child might get better treatment………………….   
   2. I want doctors to help learn more about TB………..   
5. I’ve decided to enroll my baby in the study
   1. even though my baby will receive no extra treatment   
   2. because I knew I would receive a toiletries hamper   
6. If my baby gets a bruise from the blood test, I should
   1. Contact the police………………………………………   
   2. Speak to the nurse at the clinic………………………   
   3. Go to the doctor at his private surgery………………   

16. I was enrolled in the study in my home language…………..   

17. If I was given the choice to participate again, I would.…….   

1. Which of the following rights are protected in the

South African constitution?

(a) access to health care…............…….…………………….   

(b) concealment of private information……………………….   

(c) freedom from bodily harm...........……………………….   

(d) free health care to all……............……………………….   

(e) freedom of choice….……............……………………….   

| If contradictory or exploratory comments were made, please note the question number and record the details in the adjacent space. Also ask if there are any questions and record details. | |
| --- | --- |
|  |  |
|  |  |
|  |  |
|  |  |

| **Question** | **Answer** |  | **Correct?** | **Question** | **Answer** |  | **Correct?** |
| --- | --- | --- | --- | --- | --- | --- | --- |
| **1** |  | **B** |  | **13b** |  |  | |
| **2** |  | **A** |  | **14a** |  |
| **3** |  | **B** |  | **14b** |  |
| **4** |  | **C** |  | **15a** |  |
| **5** |  | **B** |  | **15b** |  |
| **6** |  | **A** |  | **15c** |  |
| **7** |  | **C** |  | **16** |  | **Yes** |  |
| **8** |  | **A** |  | **17** |  |  | |
| **9** |  | **C** |  | **18a** |  | **Yes** |  |
| **10a** |  | **Yes** |  | **18b** |  | **No** |  |
| **10b** |  | **No** |  | **18c** |  | **Yes** |  |
| **11a** |  | **Yes** |  | **18d** |  | **No** |  |
| **11b** |  | **No** |  | **18e** |  | **Yes** |  |
| **12** |  | **No** |  |  |  |  |  |
| **13a** |  |  | | **Total** | | |  |

The length of time it took to complete the questionnaire was:

- - 1. Under 10 minutes
    2. Between 10 and 20 minutes
    3. Longer than 20 minutes

The amount of assistance given by study staff to complete the questionnaire was:

1. Minimal, only clarifications required here and there
2. Moderate
3. Substantial, the participant required much help

| Name of Consenter of Case Control Study |  |
| --- | --- |

______________________ ______________________

(Signature of research nurse) (Date)

_______________________ ______________________

(Signature of data capturer) (Date)
